# Does antihypertensive treatment with renin-angiotensin system inhibitors prevent the development of diabetic kidney disease?

**DOI:** 10.1186/s40360-015-0024-y

**Published:** 2015-09-11

**Authors:** Hiroki Miyazaki, Akira Babazono, Takumi Nishi, Toshiki Maeda, Takuya Imatoh, Masayoshi Ichiba, Hiroshi Une

**Affiliations:** Department of Health Care Administration and Management, Graduate School of Medical Sciences, Kyushu University, 3-1-1 Maidashi, Higashi-ku, Fukuoka, 812-8582 Japan; Department of Social and Environmental Medicine, Faculty of Medicine, Saga University, Saga, Japan; Department of Hygiene and Preventive Medicine, Faculty of Medicine, Fukuoka University, Fukuoka, Japan; Medical Research Center, Faculty of Medicine, Fukuoka University, Fukuoka, Japan

## Abstract

**Background:**

Diabetic kidney disease (DKD) is the leading cause of end-stage renal disease worldwide. Renin-angiotensin system (RAS) inhibitors are the first-line treatment for diabetic patients with hypertension. However, whether RAS inhibitors prevent the development of DKD remains controversial. We conducted a retrospective cohort study quantifying the preventive effect of antihypertensive treatment with RAS inhibitors on DKD, using data from specific health check-ups and health insurance claims.

**Methods:**

The study subjects were 418 patients with diabetes and hypertension, drawn from health insurance societies located in Fukuoka and Shizuoka prefectures in Japan. The subjects were divided into three groups, according to the type of antihypertensive treatment they received. They were then compared in terms of the development of DKD, using the diagnostic codes from ICD-10.

**Results:**

Thirty subjects (6.2 %) developed DKD during the study period between April 2011 and September 2013. RAS inhibitor treated group showed a significantly lower risk of DKD [adjusted odds ratio (AOR) = 0.35; 95 % confidential interval (CI): 0.16–0.76] compared with the no treatment group.

**Conclusion:**

We conclude that antihypertensive treatment with RAS inhibitors is potentially useful for preventing the development of DKD.

## Background

Diabetes mellitus (DM) is the leading cause of chronic kidney disease (CKD) worldwide [1]. Defined as having a decreased glomerular filtration rate or elevated urine albumin excretion level, CKD is a major risk factor for cardiovascular diseases as well as end-stage renal disease (ESRD) [2]. The prognosis for diabetic patients is worsened when complicated with CKD. Therefore, special attention has recently been paid to this complication, defined as diabetic kidney disease (DKD) [3, 4]. The progression of DKD is associated with substantial morbidity, mortality, and costs. Therefore, its prevention and management have been increasingly recognized as being important for clinical care, disease management, and public health.

Although the mechanism underlying the development of DKD is still unknown, it is widely accepted that the treatment of hypertension is as important as optimal glycemic control in preventing the development of DKD among diabetic patients [5]. Many studies have demonstrated that renin-angiotensin system (RAS) inhibitors such as angiotensin receptor blockers (ARBs) and angiotensin converting enzyme inhibitors (ACE-Is) slow the rate of progression of DKD and reduce the incidence of cardiovascular disease and ESRD [6–9]. However, whether RAS inhibitors prevent the development of DKD remains controversial [10–14].

Currently, the guidelines for the management of hypertension in Japan [15], as well as the United States [16, 17] and Europe [18], recommend RAS inhibitors as first-line treatment for diabetic patients with hypertension. However, the proportion of RAS inhibitors used clinically in diabetic patients remains unclarified. Moreover, the degree to which RAS inhibitors reduce the risk of DKD has not been fully investigated. Therefore, we conducted a retrospective cohort study quantifying the effect of antihypertensive treatment with RAS inhibitors on the prevention of DKD, using specific health check-ups and health insurance claims data.

## Methods

### Data sources

**Fig. 1:**
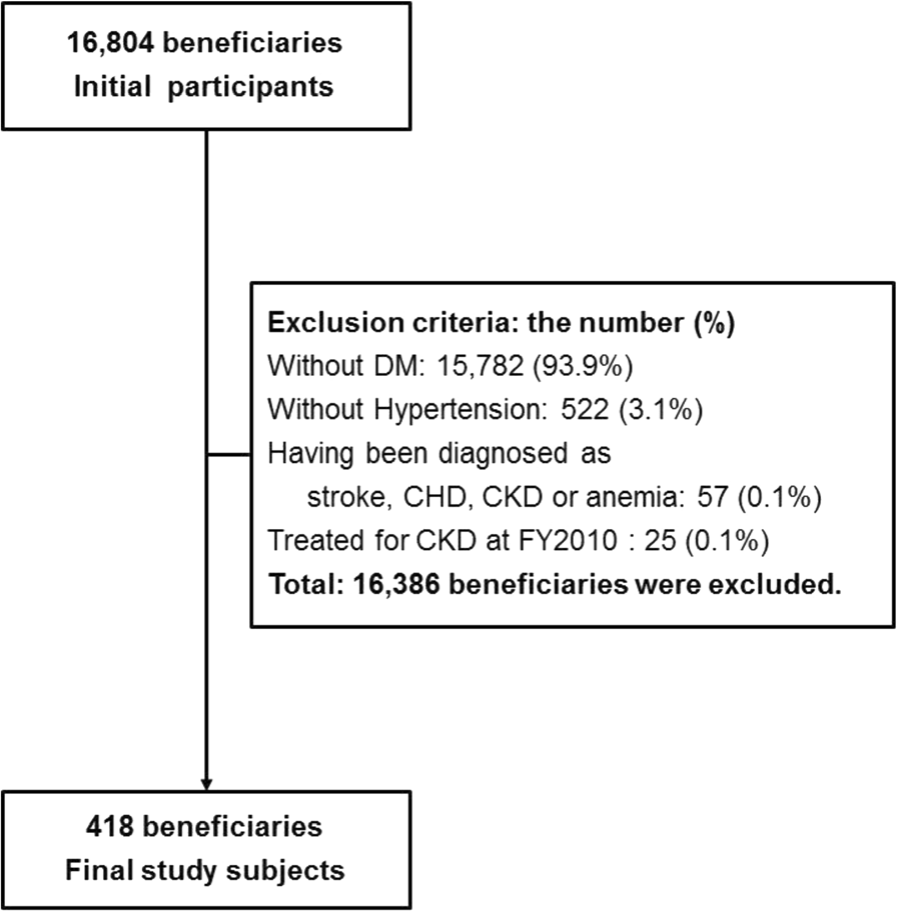
Inclusion and exclusion flowchart. DM; diabetes mellitus, CHD; coronary heart disease, CKD; chronic kidney disease

We identified 16,804 beneficiaries aged ≥ 40 years as of March 31, 2011, who worked for health insurance societies located in the Japanese prefectures of Fukuoka and Shizuoka, and attended specific health check-ups in the 2010 fiscal year (FY). From this population, we identified 1022 subjects whose hemoglobin A1c (HbA1c) level [taken from the national glycohemoglobin standardization program (NGSP)] was ≥ 6.5 %, and/or who were receiving treatment for diabetes. Next, we excluded 522 subjects without hypertension. Subjects with hypertension were identified as those using antihypertensive drugs and/or having a systolic blood pressure (SBP) of ≥140 mmHg or diastolic blood pressure (DBP) of ≥90 mmHg. Blood pressure was determined from the average of two consecutive measurements, separated by 30 s and after 5 min of rest. Owing to the nature of the health checkup, blood pressure was only measured on the day of the checkup. We also excluded 57 subjects diagnosed as having stroke, chronic heart disease, CKD, or anemia, and 25 subjects whose health insurance claim data indicated a diagnosis of kidney disease between January and March 2011. Unfortunately, as this retrospective study was conducted using administrative data, only those with a diagnosed kidney disease were excluded. Finally, we obtained 418 study subjects (Fig. 1). The study was in compliance with the Helsinki Declaration and approved by the Kyushu University Institutional Board for Clinical Research (25–340).

### Main outcome

The main outcome in this study was the development of DKD. DKD was defined as diabetes with the presence of albuminuria, decreased GFR, or both. We identified patients with DKD between April 2011 and September 2013 on the basis of the diagnostic codes from the International Classification of Diseases, 10th Revision (ICD-10) (Table 1).

**Table 1:** Definition of diabetic kidney disease and International Classification of Diseases, 10th revision

Code	Description
ICD E102	Type 1 DM with incipient diabetes nephropathy
ICD E112	Type 2 DM with incipient diabetes nephropathy
ICD E142	Unspecified DM with incipient diabetes nephropathy
ICD N083	Glomerular disorders in diabetes mellitus
ICD N180	End-stage renal disease
ICD N189	Chronic kidney disease, unspecified
ICD I129	Hypertensive chronic kidney disease

### Definition of variables

Subjects whose HbA1c values were ≥6.5 %, or who were receiving diabetic treatment in FY 2010 were defined as having DM. Ages were categorized into three groups: 40–49 years, 50–59 years, and ≥60 years. Baseline HbA1c levels were categorized into three groups: ≤7.0, 7.1–8.0, and ≥8.1.

We divided the subjects into three groups, according to the type of antihypertensive treatment and compared the groups in terms of the development of DKD after 2.5 years of follow-up. The ‘no treatment’ group included hypertensive patients who were untreated. The RAS treatment group included those who had received antihypertensive treatment with RAS inhibitors. The RAS inhibitors included ARBs and ACE-Is. Although the direct renin inhibitor is also categorized as a RAS inhibitor, none of the patients received the drug in this study. The non-RAS treatment group included those who had received treatment other than RAS inhibitors such as calcium channel blockers, beta blockers, alpha blockers, and diuretics.

### Statistical analyses

Subject characteristics were summarized using frequencies and proportions for categorical variables and median and interquartile ranges for continuous variables. The categorical variables were compared between the three groups using Pearson’s chi-square test, and the continuous variables were compared between the three groups using the Kruskal-Wallis test.

Multiple logistic regression analyses were carried out to estimate adjusted odds ratios (AORs) and 95 % confidence intervals (CIs) for the development of DKD. We set the development of DKD as the dependent variable, and categorized age, gender, HbA1c level, prefecture, and treatment type for hypertension as independent variables.

Statistical analyses were performed using IBM SPSS Statistics for Windows, Version 20.0 (IBM Corp., Armonk, NY, USA). *P* values <0.05 were regarded as statistically significant.

## Results

We identified 136 patients in the no treatment group, 11 patients in the non-RAS treatment group, and 271 patients in the RAS treatment group. Baseline characteristics are presented in Table 2, stratified according to the type of antihypertensive treatment. There were no significant differences in age, age category, gender, prefecture, or median BMI among the three groups. Distributions of HbA1c level, SBP, and DBP were significantly different among the three groups. Additionally, there were no significant differences in the proportions of subjects with hypercholesterolemia and the smoking.

**Table 2:** Subject characteristics according to treatment groups

		Total	No treatment	Non-RAS treatment	RAS treatment	*P*-value
		(*N* = 418)	(*N* = 136)	(*N* = 11)	(*N* = 271)	
Median age	[IQR]	53[9]	53[11]	52[17]	53[9]	0.944^a^
Age	40–49	103(25 %)	40(29.4 %)	3(27.3 %)	60 (22.1 %)	
	50–59	233(56 %)	70(51.5 %)	4(36.4 %)	159(58.7 %)	0.268
	60≤	82(20 %)	26(19.1 %)	4(36.4 %)	52(19.2 %)	
Gender	Male	361(86 %)	122(89.7 %)	9(81.8 %)	230(84.9 %)	0.369
	Female	57(14 %)	14(10.3 %)	2(18.2 %)	41(15.1 %)	
Prefecture	Fukuoka	198(47 %)	72(52.9 %)	6(54.5 %)	121(44.6 %)	0.315
Median BMI, kg/m^2^	[IQR]	26.1[4.6]	26.2[44]	24.8[4.2]	26.1[4.6]	0.302^a^
Median WC, cm	[IQR]	90.3[13]	90.2[12.5]	87.5[10]	90.5[12.5]	0.368^a^
Median SBP, mmHg	[IQR]	142[19]	146[12]	139[31]	138[22]	0.001^a^
Median DBP, mmHg	[IQR]	88[14]	92[10]	82[20]	84[12]	0.001^a^
Median HbA1c, %	[IQR]	7.1[1.5]	7.4[1.8]	6.5[0.6]	7.1[1.5]	0.001^a^
Median TGs, mg/dl	[IQR]	139[106]	141.5[44]	139[73]	137[101]	0.94^a^
Median GGT, U/I	[IQR]	54[58]	46[49]	44[76]	57[57]	0.153^a^
Hypercholesterolemia		734(33 %)	337(30.4 %)	143(34.2 %)	151(32.7)	0.389
Smoking		493(22 %)	211(19 %)	93(22.2 %)	122(26.4)	0.821

Numbers are median [IQR] or number (%), unless otherwise stated. *IQR* interquartile range

*BMI* body mass index, *WC* waist circumference, *TGs* triglycerides, *GGT* gamma-glutamyltransferase

^a^Compared using the Kruskal-Wallis test. Other comparisons made using Pearson's chi-square test

During the follow-up period, 16 (11.8 %), 2 (22.2 %), and 12 (4.4 %) subjects developed DKD in the no treatment, non-RAS treatment, and RAS treatment groups, respectively. Table 3 shows the results of multiple logistic regression analyses. The RAS treatment group had a significantly lower risk of DKD (OR = 0.35; 95 % CI 0.16–0.76, AOR = 0.37; 95 % CI: 0.16–0.83) than the no treatment group. In contrast, the non-RAS treatment group had a higher risk of DKD (OR = 1.67; 95 % CI: 0.33–8.41, AOR = 2.40; 95 % CI: 0.43–13.48) than the no treatment group, although this difference was not statistically significant.

**Table 3:** Crude and adjusted odds ratios for DKD development

	Total	Subject developed DKD	Subject did not develop DKD	Unadjusted (95 % CI)	Adjusted^a^ (95 % CI)	*P*-value
	(*N* = 418)	(*N* = 30)	(*N* = 388)			
No treatment	136(100 %)	16(11.8 %)	120(88.2 %)	1.00(reference)	1.00(reference)	
Non-RAS treatment	11(100 %)	2(18.2 %)	9(81.8 %)	1.67(0.33 to 8.41)	2.40(0.43 to 13.48)	0.366
RAS-treatment	271(100 %)	12(4.4 %)	259(95.6 %)	0.35(0.16 to 0.83)	0.37(0.16 to 0.83)	0.015

^a^Adjusted by age, gender, prefecture, and HbA1c

Hosmer-Lemeshow goodness of fit: *P* = 0.151

## Discussion

Our results indicated that among diabetic patients with hypertension, antihypertensive treatment with RAS inhibitors significantly reduced the risk of developing DKD. To the best of our knowledge, this is the first study using the combination of data from specific health check-ups and health insurance claims to evaluate the preventive effect of RAS treatment on DKD.

Our results are consistent with previous studies showing that RAS inhibitors reduced the likelihood of developing DKD among diabetic patients with hypertension [8, 14]. It is assumed that RAS inhibitors prevent the development of DKD by improving the hemodynamics of the kidney via inhibition of angiotensin II overactivity [19].

RAS inhibitors were used for 96 % of the antihypertensive-treated group, indicating a high rate of compliance with the guidelines. However, 32.5 % of all the subjects with hypertension were not treated for hypertension, despite the fact that diabetic patients with hypertension are at the highest risk of cardiovascular events [20]. The reasons for this are not well understood. One possible reason is that the subjects with hypertension in this study may have included patients not actually hypertensive, but with a one-off abnormal blood pressure reading. A low hypertension treatment rate among diabetic patients has been similarly reported [21, 22]. Turchin et al. suggested that shorter time intervals between physician–patient encounters are associated with improved blood pressure control [23]. In terms of disease management in the future, a survey of consultation rates of the patients, including oral examination, would be necessary.

In June 2013, the Japanese government announced a ‘data health plan’ to be started in FY 2015. This plan will encourage all insurers to engage in disease prevention and health promotion programs based on administrative data such as data from health insurance claims and specific health check-ups. The Ministry of Health, Labour and Welfare will play a central role in promoting the project. Administrative data should be analyzed to monitor patient compliance and adherence to treatment, and to determine whether medical treatment is based on the guidelines. Furthermore, in terms of disease management, health guidance by insurers may play an important role in encouraging consultations.

The non-RAS treatment group showed a higher prevalence of DKD than the no treatment group, although this difference was not statistically significant. The finding could be related to the type of antihypertensive drug that they received; as in both cases, the subjects received antihypertensive drugs with diuretics. It has been suggested that diuretics cause DKD via the deterioration of glycemic control.

This study has several limitations. First, we used administrative data; thus, the confirmation of the development of DKD depended on claims data, rather than clinical data such as urine and blood test results. Second, in this study, the regularity of patient visits and drug compliance were not evaluated; these factors could be related to the development of DKD. Third, the types of RAS inhibitor were not considered. Several studies have demonstrated that each RAS inhibitor has a different mode of action and displays properties that may or may not be replicated by any other RAS inhibitor [13, 24]. In this regard, further studies are necessary to clarify the details of RAS treatment, namely, ARB, ACE-I, or combination therapy. Finally, the homogeneity of the study group may limit the generalizability of our findings to other racial and ethnic groups. Further evidence is needed to corroborate our findings in other populations.

## Conclusion

This study suggests that antihypertensive treatment with RAS inhibitors is potentially useful for the prevention of DKD among diabetic patients with hypertension.

## Abbreviations

DM: Diabetes mellitus
CKD: Chronic kidney disease
GFR: Glomerular filtration rate
DKD: Diabetic kidney disease
RAS: Renin-angiotensin system
BMI: Body mass index
ICD: International Classification of Diseases
ESRD: End-stage renal disease
HbA1c: Hemoglobin A1c
FY: Fiscal year
NGSP: National glycohemoglobin standardization program
